# Hysteroscopic Confirmation of a Uterine Arteriovenous Malformation as a Possible Cause of Recurrent Pregnancy Loss

**DOI:** 10.1155/crog/6655067

**Published:** 2025-07-25

**Authors:** Lionel Reyftmann, Dharmesh Kothari, Mark Power

**Affiliations:** ^1^Genea, Wollongong Day Surgery, Wollongong, New South Wales, Australia; ^2^Graduate Medicine, University of Wollongong, Wollongong, New South Wales, Australia; ^3^Wollongong Obstetrics & Gynaecology, Wollongong Day Surgery, Wollongong, New South Wales, Australia; ^4^Department of Interventional Radiology, Wollongong Hospital, Wollongong, New South Wales, Australia

**Keywords:** abortion, habitual [C12.050.703.039.089], arteriovenous malformations [C14.240.850.750], hysteroscopy [E01.370.388.250.360], radiography, interventional E01.370.350.700.725

## Abstract

This case report highlights the cornerstone role played by hysteroscopy to confirm a diagnosis of uterine arteriovenous malformations that was ambiguous with the imaging studies. A 30-year-old nulliparous woman who experienced three unexplained recurrent pregnancy losses was suspected of having a uterine arteriovenous malformation. The arteriovenous malformation was confirmed through hysteroscopy and managed with a multidisciplinary approach involving interventional radiology and reproductive specialists. The hysteroscopy was followed by uterine artery embolization, which resulted in the resolution of the arteriovenous malformation. A spontaneous pregnancy and live birth rapidly followed. Uterine arteriovenous malformations have been widely reported in gynecology as a consequence of the surgical treatment of a miscarriage or gestational trophoblastic disease. We suggest that they are also important to diagnose in patients presenting with recurrent pregnancy loss, where they represent a curable etiology.

## 1. Introduction

Recurrent pregnancy loss (RPL) is defined as the occurrence of two or more consecutive spontaneous pregnancy losses before 24 weeks of gestation [1]. Although RPL has various causes, uterine arteriovenous malformations (AVMs) are a rare but noteworthy etiology [2, 3]. Uterine AVMs involve abnormal vascular connections between arteries and veins in the uterine wall, leading to impaired blood flow. This case report describes how hysteroscopy can refine the diagnosis and improve the management of a patient with a uterine AVM presenting with RPL.

## 2. Case Report

A 30-year-old nulliparous pregnant woman presented to the reproductive medicine clinic at a gestational age of 8 weeks. She had a history of two consecutive spontaneous abortions at 5 and 8 weeks, respectively. The second miscarriage was hemorrhagic and required a D and C and the placement of a Foley catheter for uterine tamponade. A possible uterine AVM had been suggested by the ultrasound in a regional center, but unfortunately, this was not investigated with an MRI or a CT angiogram.

**Figure 1:**
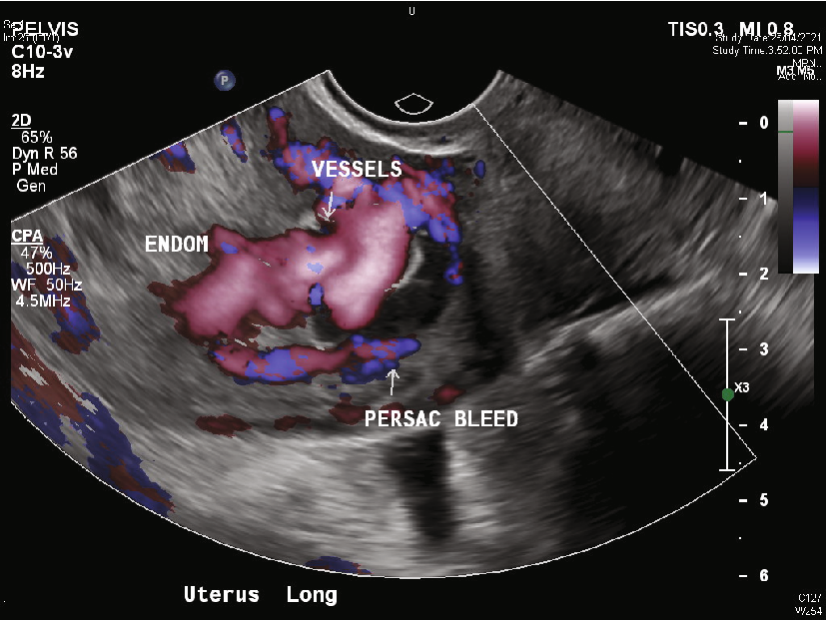
Transvaginal ultrasound suspicious for a uterine AVM.

**Figure 2:**
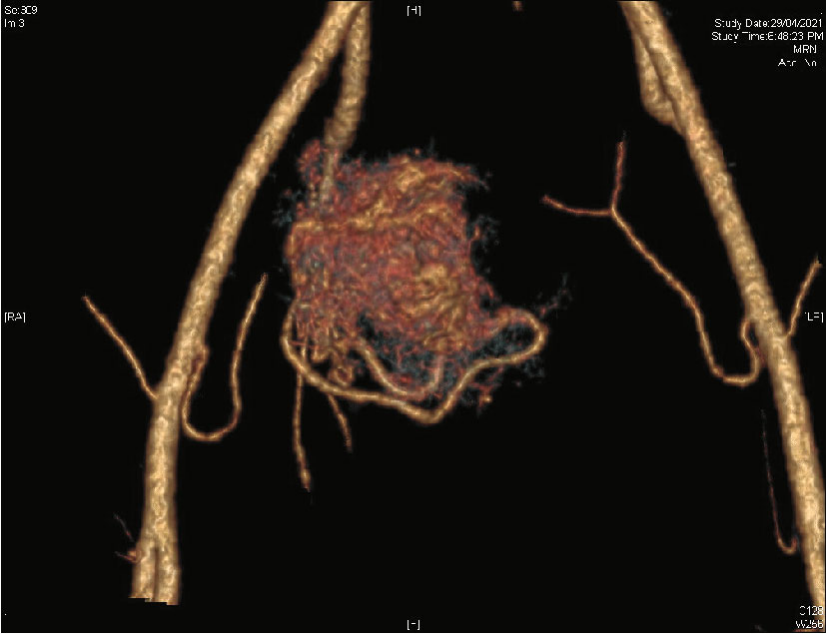
Contrast-enhanced CT angiogram showing the right uterine artery feeding the AVM.

This third pregnancy was confirmed to be nonviable on the bedside ultrasound. Transvaginal ultrasound revealed a 2.3 cm sac with a small subchorionic hemorrhage, as well as a highly vascularized right side of the myometrium, suspicious for a uterine AVM (Figure 1). Subsequently, a contrast-enhanced CT angiogram was performed to confirm the diagnosis and assess the extent of the malformation (Figure 2). Although the radiologist's diagnosis confirmed the presumption of an AVM, the obstetrical team suggested a simple increased vascularity caused by the physiological changes related to the pregnancy. The patient was counselled about medical and surgical options for the evacuation of the uterus and the risk of bleeding in both cases. As she lived 70 km away from our hospital, she opted for a surgical treatment under general anesthesia.

A surgical evacuation was organized in the operative theatre under ultrasound guidance, and care was taken to avoid direct suction of the right uterine wall. The procedure was uncomplicated, and a preventive Foley catheter was left in the cavity for 4 h and progressively deflated without further bleeding. The histopathology confirmed normal products of conception without gestational trophoblastic disease, but most importantly, the microarray analysis returned normal: (1–22, X) ×2. Given the RPL and the suspicion of AVM, further evaluation was pursued. Initial investigations for common causes of RPL, including genetic, hormonal, and immunological factors, were unremarkable (parental karyotypes 46 XX and 46XY, low vaginal swab, antinuclear antibodies, screening for antiphospholipid syndrome, homocysteinemia, TSH, glycaemia, and HbA1c).

**Figure 3:**
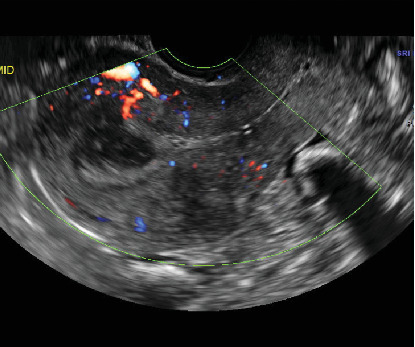
Postabortum transvaginal ultrasound showing resolving retained products of conception and low vascular flow inconsistent with AVM.

The postabortum ultrasound showed resolving retained products of conception and stated that low vascular flow was not consistent with an AVM (Figure 3).

As the sonography was discordant with the clinical history, we made the decision to investigate the cavity with a hysteroscopy. The procedure was conducted under general anesthesia in the theatre due to the potential risk of bleeding. We visualized a bluish vascular mass in the right anterior wall that was not bleeding or pulsatile but clearly protruded in the cavity as soon as a controlled pressure drop was achieved (Video S1). To exclude other causes of RPL, and despite the theoretical risk of bleeding, a careful biopsy of the endometrium was taken on the left side of the cavity, away from the nidus. It ruled out chronic endometritis and documented normal populations of macrophages and natural killer cells.

A multidisciplinary team consisting of reproductive medicine specialists and interventional radiologists was involved in the management of the patient. Given the potential risks associated with pregnancy continuation in the presence of a uterine AVM, the decision was made to treat the malformation prior to attempting conception again.

**Figure 4:**
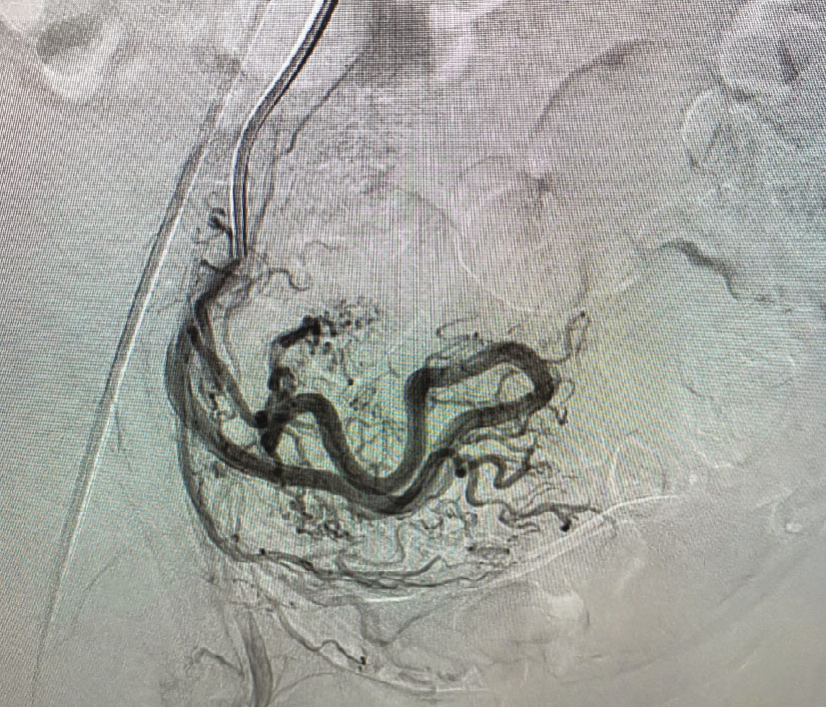
Angiogram with selective catheterization of the right uterine artery.

**Figure 5:**
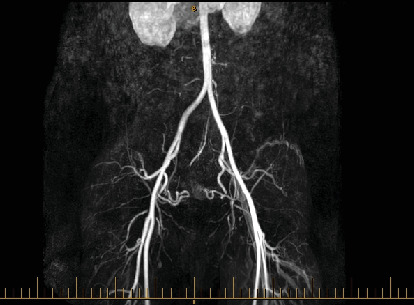
Follow-up angio-MRI demonstrating the regression of the uterine AVM.

The patient underwent transcatheter arterial embolization (TAE) under fluoroscopic guidance. Selective catheterization of the uterine artery was performed, and porcine sponge gelatine (gel foam⁣^∗^) was injected to occlude the abnormal vessels supplying the AVM (Figure 4). The procedure was successful, and the patient tolerated it well, with no immediate complications. Postembolization, the follow-up angio-MRI examination demonstrated regression of the uterine AVM, with a significant reduction in vascularity (Figure 5). Once the vascular architecture was stabilized, the patient was advised to attempt conception. The patient successfully conceived 4 months after the procedure and achieved an uneventful pregnancy. Regular antenatal care was provided, including close monitoring of the uterine vasculature. The patient delivered a 3200 g healthy male infant via emergency C-section at 38 weeks for fetal distress after SROM and failed IOL. The patient expressed a great satisfaction with the positive outcome and provided informed consent for the publication of her case.

## 3. Discussion

Uterine AVMs are rare entities but should be considered in the differential diagnosis of RPL [2–4]. Although histopathology is arguably the gold standard for confirming the diagnosis of AVM (a proliferative vascular nidus characterized by arteriovenous shunts, predominantly composed of dilated vascular structures that are thin-walled and sometimes thickened), imaging modalities such as transvaginal ultrasound with color Doppler and contrast-enhanced angiogram or MRI play crucial roles in confirming the diagnosis and assessing the extent of the malformation. The Doppler finding of a high-velocity, low-impedance flow in a vascular structure exhibiting shunts with higher peak velocity generally signifies the diagnosis, but some authors have suggested a role for hysteroscopy to confirm the diagnosis and assist with follow-up [5]. The originality of our presentation resides in the central role of hysteroscopic diagnostics. Importantly, hysteroscopy was offered to elucidate a diagnostic conundrum but was not intended to treat the AVM, although this has been published in the literature [6]. We speculate that a small AVM may become visible only during pregnancy due to the increased vascularity related to gestation. There is increasing evidence that uterine AVM and abnormal involution of the vessels of the placental sign may belong to a continuum, and it may not always be obvious to make a clear distinction [7, 8], which nevertheless has clinical implications. The use of intrauterine techniques, such as hysterosalpingogram [3] or sonohysterography, has been described but is generally not necessary and can be potentially dangerous (posing risk of bleeding or absorption of the contrast agent). Treatment options for uterine AVMs, such as embolization techniques, are appropriate and well supported by a strong body of literature, depending on the patient's clinical presentation and desire for future fertility [9, 10]. There is no consensus on the ideal waiting time for pregnancy after embolization when fertility is desired. However, a recent publication reported a short median time to conception of 2.5 months, which is in line with our observation [8].

## 4. Conclusion

This case report highlights the significance of uterine AVM as a potential cause of RPL and how hysteroscopy can help to reveal subtle cases. Prompt diagnosis and appropriate management, involving a multidisciplinary team, can lead to successful treatment and subsequent healthy pregnancies. Although this seems a rare entity, clinicians should be aware of uterine AVMs when evaluating patients with RPL, especially when routine investigations yield no identifiable cause.

## Data Availability

The data that support the findings of this study are available from the corresponding author upon reasonable request.

## Supporting Information

Supporting Information Additional supporting information can be found online in the Supporting Information section. Video S1: Hysteroscopic view of the AVM. This video shows a hysteroscopic view of the right-sided AVM confirming the final diagnosis. It also demonstrates the visually guided endometrial biopsy on the left side of the cavity.
